# Antimicrobial photodynamic chemotherapy mediated by PapaMBlue on chronic periodontal disease: Study protocol for a randomized, blind, controlled trial

**DOI:** 10.1097/MD.0000000000018854

**Published:** 2020-02-07

**Authors:** Giuliana Giovinazzo Anselmo, Ana Carolina Alves Camargo Tortamano, Marcela Letícia Leal Gonçalves, Adriana Leal-Rossi, Bianca Aparecida Godoy-Miranda, Márcia Regina Cabral Oliveira, Pedro Henrique Cabral Oliveira, Carol Brandt Alves, Sandra Kalil Bussadori, Renato Araujo Prates

**Affiliations:** a Post Graduation Program in Biophotonics Applied to Health Sciences Universidade Nove de Julho; b Department of Dentistry, School of Dentistry, Universidade Nove de Julho (UNINOVE), São Paulo, Brazil.

**Keywords:** antimicrobial photodynamic therapy, laser, periodontitis, photosensitizer

## Abstract

**Background:**

The elimination of the pathogenic microorganisms of the periodontal pocket is one of the main points for success in periodontal treatment. The objective of this study is to investigate the clinical and antimicrobial effect of papain-mediated photodynamic therapy in the clinical treatment of periodontal disease.

**Methods:**

Twenty patients with chronic periodontitis will be selected. Patients will be randomly divided into 2 groups (n = 10). Group 1 will receive conventional periodontal treatment and group 2 will receive conventional treatment and antimicrobial photodynamic therapy (PACT). Conventional treatment will consist of oral hygiene guidance, with brushing technique instructions and recommendation of daily flossing. The calculus deposits on the teeth will be removed with ultrasound equipment and curettes for scraping and root planning. The PACT will be performed at the end of each periodontal treatment session, at sites with bags ≥4 mm. PapaMblue photosensitizer will be deposited in the periodontal pockets with a syringe and a pre-irradiation time of 1 minute will be adopted. Then, the laser emitting wavelength of 660 nm, with power of 100 mW, for 2 minutes, radiant exposure of 30 J/cm^2^ and power density of 250 mW/cm^2^ will be applied. Patients will undergo clinical evaluations before treatment (day 1) at 30, 60, and 90 days after the end of treatment; and microbiological evaluations before and immediately after treatment. The distribution of the data within each group and the homogeneity of the variances will be verified. With this information, the most appropriate statistical test in each evaluation will be used. The sample calculation is based on the literature and the significance level of 5% will be adopted.

**Discussion:**

The combination of PACT with methylene blue in a papain gel and the conventional treatment may increase the reduction of bacteria in periodontal pockets.

## 1 Introduction

Periodontitis is an inflammatory disease of supporting tissues of the teeth, and it is induced by specific microorganisms and influenced by the immune-inflammatory response of the host. It is characterized by a microbiological association and inflammation that results in loss of the alveolar bone or sustained periodontium. Bleeding is noted when the clinical depth of probing of the periodontal sacs is measured; evident sign of active periodontal disease. The epithelial tissue does not present vascularization, but connective tissue, indicating invasion by periodontopathogens, thus triggering an inflammatory immune response because of bacterial invasion.^[1–3]^

According to the new classification of periodontal diseases, periodontitis is characterized by loss of insertion, alveolar bone resorption, loss of periodontal ligament fibers and root cement contamination. The main factor responsible for periodontitis is the association between strict anaerobic Gram negative bacteria within the periodontal pocket. It also has inflammation in the gingival connective tissue, with a predominance of plasma cells and lymphocytes, there is an increase of proinflammatory cytokines in the site, contributing to tissue destruction and disorganization. The nutrition of the gingival epithelium is also compromised. Thus, cells of the junctional epithelium proliferate and migrate in the apical direction, the main characteristic of the formation of periodontal pockets.^[4]^

The goal of periodontal treatment is to eliminate bacterial deposits by removing the supra and subgingival biofilm, and consequently reducing the excessive inflammatory response. The procedures involve diagnostic steps, followed by biofilm removal and biofilm associated calculation followed by control and maintenance, with the control of etiological agents and risk factors, based on clinical outcomes with non-surgical treatment approaches with the scraping and straightening of the corono-root with manual and ultrasonic instruments, which in addition to the removal of biofilm deposits, contributes to the reduction and modification of biofilm colonizing periodontopathogens. It is noteworthy that patient compliance and contribution is extremely important in the control of health stability in periodontal tissues. Assuming that when biofilm is controlled, it does not accumulate and colonize with more periodontopathogenic species, thus not inducing an inflammatory response related to periodontal disease. The use of lasers for periodontal treatment in noninvasive therapies presents a review of the current state of evidence for the efficacy of these approaches as an adjunctive treatment to mechanical biofilm deposition removal therapy.^[5]^

Photodynamic antimicrobial chemotherapy (PACT) involves the use of a photosensitizer (PS) that is activated by light at resonant wavelength. This phenomena populate the triplet state of the photosensitizer, which at this moment may transfer energy to the medium's oxygen and results in the formation of reactive oxygen species (ROS) such as singlet oxygen, superoxide anion, hydroxyl radical, and hydrogen. These ROS can damage proteins, lipids, nucleic acids, and other cellular components.^[6]^ PACT is a noninvasive modality of treatment, and can be used as an adjuvant to the treatment of periodontitis. For the periodontal treatment, PS binds to the cells of the oral biofilm, and when activated by light they promote the physical disorganization of the biofilm and death of microorganisms by the formation of ROS, and other factors, such as the presence of a high level of metabolic activity.^[7]^

The effects of PACT on periodontal disease has been investigated as an adjuvant treatment to periodontitis and one class of photosensitizers frequently used is the blue dyes of the phenothiazines family, among them toluidine blue and methylene blue. Phenothiazines are flat tricyclic molecules that have a quaternary nitrogen atom and have efficient phototoxicity against various microorganisms. Currently, phenothiazines represent the only photosensitizers employed clinically in antimicrobial treatment.^[6]^ In an in vitro study performed by Alvarenga et al,^[7]^ PACT was evaluated using PACT mediated by 100 μM methylene blue and laser irradiation emitting 660 nm with 100 mW of output-light power on an in vitro biofilm of *Aggregatibacter actinomycetemcomitans*, a pathogen strongly associated with periodontal disease. Three irradiation times of 1, 3, and 5 minutes were tested. The results of the study indicate that irradiation time impact on cell death, and the best result was found following 5 minutes of irradiation, in which a bacterial reduction of 99.85% was achieved.^[7]^

Despite the good results of PACT in vitro^[7,8]^ and in animal model^[9]^ the use of PACT as a complement to periodontal treatment in clinical studies has been shown to be less significant. This lack of clinical consensus can be attributed to physical, chemical, and biological characteristics related to the clinical use of PACT.

The parameters of irradiation and the behavior of the photosensitizer in the oral environment are extremely important to achieve photochemical effects and consequent microbial death. It is important to note that cellular toxicity occurs only when the PS absorption spectrum and the emitted radiation are compatible. The wavelength and intensity of the light, the exposure time, and the absorption capacity of the PS determine the results. The efficacy of the treatment depends on the optimization of a large number of parameters,^[10]^ therefore, there is a need to study new photosensitizers with greater stability for clinical use. In order to evaluate the effect of the presence of ROS on the formation of dimers, the results of this study are presented in Table 1. PapaMBlue is already used clinically with positive results in the removal and disinfection of carious lesions. This randomized, blinded trial aims to evaluate the use of PapaMBlue as a mediator of PACT in patients with chronic periodontitis.

**Table 1:**
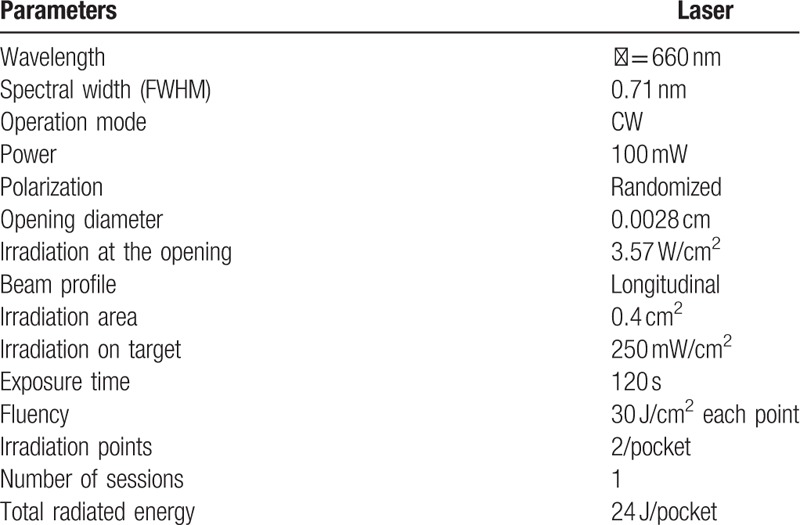
Laser parameters.

In 2003, a Brazilian formulation was launched in the market called “Papacárie” (Fórmula e Ação, São Paulo, SP, Brazil). The product is a papain based and chloramine gel. This product was presented as an alternative to facilitate the chemical and mechanical removal of dental caries. This gel acts only on infected tissue due to the absence of a plasma antiprotease, alpha 1-antitrypsin, which prevents its proteolytic action in normal tissues. According to the manufacturer, the product facilitates the removal of caries, preserving healthy tissue, can be used without local anesthesia and use of rotary instruments and is indispensable for special patients, such as babies, children, adolescents, and the elderly.^[11,12]^

Papacárie has papain cleansing properties combined with disinfection. Collagen fibrils exposed by the dissolution of surrounding minerals in dentin due to the action of bacteria are susceptible to interaction with the papain-based gel, allowing decayed tissue to be removed with a blunt-tipped instrument. This gel has been successfully used in clinical trials for decayed tissue removal for minimally invasive patients. Papacárie can be safely used in minimal intervention dentistry because it does not cause collagen degradation, which is essential for the dental restoration process.^[10]^

Antimicrobial photodynamic chemotherapy (PACT) is a conventional photodynamic therapy variant that has emerged in minimal dental intervention due to the need to reduce the microbiota in the oral cavity and prevent the progression of caries. PACT combines a photosensitizing agent and an appropriate light wavelength at the maximum absorption peak of the photosensitizer to produce reactive oxygen species that are toxic to bacterial cells. To combine the advantages of papain gel and antimicrobial photodynamic chemotherapy, a change in the composition of Papacárie was made so that this product could be used to remove infected dentin tissue and simultaneously as an antimicrobial agent. The change involved the addition of methylene blue, which is a well-known photosensitizer activated by red light (660–10 nm), giving rise to PapaMBlue. This low cost innovation has been used in an experimental study with positive results. By preventing and treating caries to the best of their ability, dentists can minimize tooth loss and improve patients’ quality of life.^[10,13]^

The aim of this study is to investigate the effect of PapaMBlue-mediated antimicrobial photodynamic therapy (PACT) on the clinical treatment of periodontal disease.

## 2 Methods/Design

### 2.1 Type of study

This parallel group, controlled clinical trial will be performed with patients with periodontitis who are in attendance at the Nove de Julho University Dental Clinic, São Paulo, Brazil, or are referred for treatment at this center, where all clinical procedures for the study will be performed. It is already approved by the Ethics Committee of the Nove de Julho University (68754517.5.0000.5511). Research participants should read, understand, and sign the Informed Consent Form, approved by the Nove de Julho University Human Research Ethics Committee.

**Figure 1:**
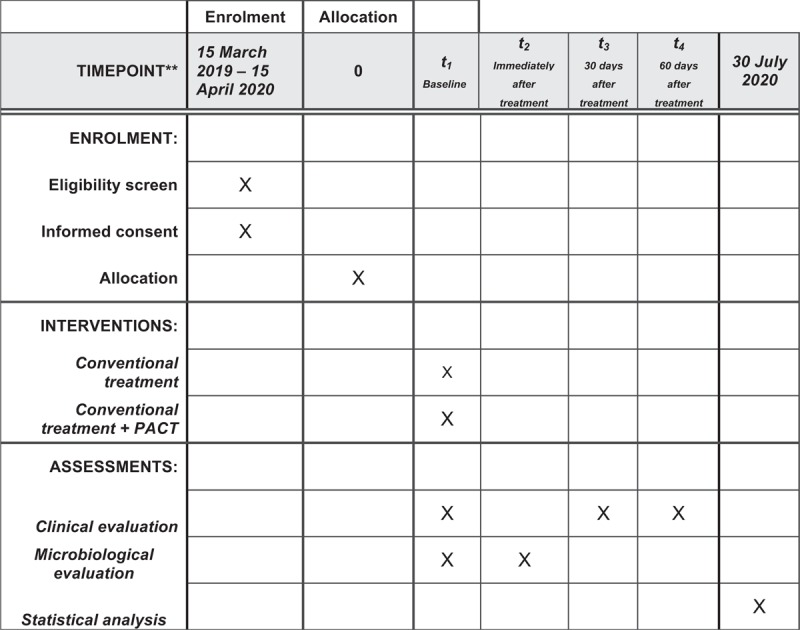
SPIRIT figure as recommended by 2013 SPIRIT statement. SPIRIT = Standard Protocol Items: Recommendations for Interventional Trials.

The protocol is in accordance with the 2013 SPIRIT (Standard Protocol Items: Recommendations for Interventional Trials) Statement. The SPIRIT checklist can be found as an additional file and Fig. 1 is the SPIRIT figure. SPIRIT was developed to provide guidance in the form of a checklist of recommended items to include in a clinical trial protocol, to help improve its content and quality.

### 2.2 Trial registration

This clinical trial was registered in ClinicalTrials.gov (NCT03855345), first posted and last updated February 26, 2019.

### 2.3 Sample calculation

Sample size was calculated based on results of 3 patients and standard deviation of bacterial survival fraction was used on the equation: 
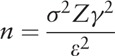
*n* is the sample size, *ε* is the maximum error (5%), *σ* is the experimental standard deviation, and Zγ is confidence interval that we expect (95%), with *Z* score at 1.96. Experimental data demonstrated that we need at least 5 patients per group to achieve a probability of 95% to be right.

### 2.4 Recruitment and randomization

Patients with periodontitis who are in attendance at the Nove de Julho University Dental Clinic or are referred for treatment at this center will be recruited. As they will already be in treatment, we expect adherence to be easier. Patients will be randomly divided into 2 groups (n = 10). Group 1 will receive conventional treatment and Group 2 will receive conventional treatment and antimicrobial photodynamic therapy (PACT). The distribution in the experimental groups will be random, and a draw will be made with numbers. As the numbers are drawn, they will make up the experimental groups. Brown envelopes will be identified with each number and, inside them, will be inserted a sheet containing the information of the experimental group, according to the order obtained in the draw. Envelopes will be sealed and remain sealed in numerical order in a safe place until the time of the procedures.

#### 2.4.1 Inclusion criteria

- The patient should have periodontitis.
- Have at least 10 teeth present, with at least ≥2 non-adjacent interproximal sites with 1 to 2 mm insertion loss. Presence of a clinical probing depth of at least 4 mm and clinical signs of inflammation, probing bleeding >10%.^[1,14–16]^
- The patient should be undergoing periodontal treatment at the Nove de Julho University Dental Clinic, institution where this research will be performed. All patients will be treated according to the protocol recommended by the American Academy of Periodontics (AAP, 2001).
- Patients with a minimum age of 18 years will be recruited.

#### 2.4.2 Exclusion Criteria

- Smokers or former smokers who discontinued the habit <12 months prior to selection;
- Uncontrolled diabetes;
- Anemia;
- Cancer;
- Pregnant women;
- Use of antibiotics in the last 6 months;
- Use of anti-inflammatory in the last 3 months;
- Coagulation disorder (use of anti-coagulant, presence of liver diseases, thrombocytopenia, and immunosuppression);
- Patients under orthodontic treatment;
- Patients who maintain a biofilm index >25%.

## 3 Evaluations and outcomes

### 3.1 Clinical examination

For evaluation of clinical parameters, a single trained and calibrated examiner will examine 4 sites per tooth (interproximal sites) with a 15 mm North Carolina millimeter probe for plaque index, bleeding on probing, clinical probing depth, gingival recession, and clinical attachment loss detected in ≥2 non-adjacent interproximal sites. Four sites per tooth (interproximal sites) will be evaluated. The evaluation will be performed at the beginning of treatment at 30, 60, and 90 days after the end of treatment. The evaluator will not know which group the patient is allocated to. Results will be considered satisfactory when at least one of the following parameters is observed:

- Primary outcome: Reduction in CFUs (colony forming units);
- Secondary outcomes: reduction in clinical depth of probing; reduction of bleeding on probing; clinical gain of insertion; reduction of biofilm index.

### 3.2 Conventional treatment

Treatment will consist of oral hygiene guidance, brushing technique instructions, and daily flossing recommendation. All patients will receive the demonstration of oral hygiene techniques. Calculus deposits on the teeth will be removed with ultrasound equipment and root scraping and straightening curettes (American Academy of Periodontology, 2001). After the use of ultrasound and curettes, a bicarbonate jet will be used to remove dental biofilm. Treatment will be performed in 2 to 4 sessions under local anesthesia (2% mepivacaine with 1:100,000 norepinephrine). Gracey periodontal curettes (numbers 3/4, 7/8, 11/12, and 13/14) and Mc Call will be used to remove dental calculi. Other biofilm retaining factors, such as carious lesions, condemned teeth, and maladaptive restorations, will be removed during these periodontal treatment sessions.

### 3.3 Antimicrobial photodynamic chemotherapy

PACT will be performed at the end of each periodontal treatment session by a second evaluator at sites with pockets ≥4 mm. The PapaMBlue photosensitizer (F&A Ltda, Sao Paulo, Brazil) will be deposited into the pouches with a syringe, with the bottom of the pouch applied in a coronal direction, and a 1-minute pre-irradiation time will be adopted so that the drug can stain. All bacterial biofilm (according to manufacturer's information). Then, the laser will be applied emitting wavelength of *ʎ* = 660 nm, with power of *P* = 100 mW. The laser will be applied to the mucosa over the oral epithelium with an optical fiber. Irradiation will be performed until the entire periodontal pocket is illuminated for 2 minutes at each spot. Each irradiation point will be of approximately 0.4 cm^2^, which will result in an energy density of 30 J/cm^2^ within 2 minutes of irradiation. The irradiation will have a constant power density of 250 mW/cm^2^. All laser parameters can be found in Table 1.

### 3.4 Microbiological evaluation

The microbiological examination will be performed from subgengival biofilm samples collected from the periodontal pockets. Two collections will be performed at each experimental site before irradiation, and immediately after the irradiation procedures. For the collection of the subgingival biofilm, the relative isolation of the teeth with cotton rollers will be done, the supragingival biofilm will be removed with sterile gauze, and the subgingival biofilm sample will be obtained by inserting a tip of sterile absorbent paper (no. 30) in the interior of the periodontal pocket, being held in place for 30 seconds. The tips will be removed and stored in properly identified sterile plastic microtubes, each tube containing 1 mL of sterile Brain Heart Infusion (BHI) culture medium will be conditioned on ice and analyzed immediately after collection.

The samples will be used to determine the CFUs (Colony Forming Units). Each tube with 1 mL BHI will be vortexed and will go through serial dilution of 10^−1^ to 10^−5^ times the original concentration. Aliquots of 10 μL in 5 dilutions will be seeded as striations on the surface of blood agar in Petri dishes. The plates will be incubated at 37 °C for a period of up to 72 hours under anaerobic conditions to evaluate recovered total bacteria. After this period, the CFU will be counted and the data will be submitted to statistical analysis.

### 3.5 Statistical analysis

The distribution of the data within each group and the homogeneity of the variances will be verified. With this information, the most appropriate statistical test in each experiment will be used. The significance level of 5% will be adopted.

## 4 Discussion

Periodontal disease may be treated by a number of strategies, such as scaling and root planing, antibiotic medication, instruction for oral hygiene, and surgical approach. PACT has been reported as an adjuvant tool to improve clinical outcomes. Methylene blue-based PACT is the most used technique, however, it presents limitations on PS concentration by dimmer formation. We intend to introduce a papain gel as a drug delivery vehicle to can may decrease PS aggregation.^[13–16]^

## Author contributions

Conceive and design the study: RAP; will perform the experiment: GGA, ACACT, ALR, BAGM, MRCO, MBB, PHCO will analyze the data: PHCO, SKB, RAP; will perform the statistical analysis: RAP; write the paper: GGA, CBA, SKB, MLLG, RAP.

Renato Araujo Prates orcid: 0000-0002-8115-9237.

## References

[1] Tonetti MS Greenwell H Kornman KS Staging and grading of periodontitis: framework and proposal of a new classification and case definition. J Periodontol 2018;89:S159–72. 29926952 10.1002/JPER.18-0006

[2] Chapple ILC Mealey BL Van Dyke TE Periodontal health and gingival diseases and conditions on an intact and a reduced periodontium: Consensus report of workgroup 1 of the 2017 World Workshop on the Classification of Periodontal and Peri-Implant Diseases and Conditions. J Clin Periodontol 2018;45:S68–77. 29926499 10.1111/jcpe.12940

[3] Ramseier CA Mirra D Schütz C Bleeding on Probing as it relates to smoking status in patients enrolled in supportive periodontal therapy for at least 5 years. J Clin Periodontol 2015;42:150–9. 25469634 10.1111/jcpe.12344

[4] Mombelli A Microbial colonization of the periodontal pocket and its significance for periodontal therapy. Periodontol 2000 2018;76:85–96. 29193304 10.1111/prd.12147

[5] Ryder MI Armitage GC Minimally invasive periodontal therapy for general practitioners. Periodontol 2000 2016;71:7–9. 27045427 10.1111/prd.12132

[6] Dai T Fuchs BB Coleman JJ Concepts and principles of photodynamic therapy as an alternative antifungal discovery platform. Front Microbiol 2012;3:1–6. 22514547 10.3389/fmicb.2012.00120 PMC3322354

[7] Alvarenga LH Prates RA Yoshimura TM Aggregatibacter actinomycetemcomitans biofilm can be inactivated by methylene blue-mediated photodynamic therapy. Photodiagnosis Photodyn Ther 2015;12:131–5. 25461964 10.1016/j.pdpdt.2014.10.002

[8] Leal CRL Alvarenga LH Oliveira-Silva T Antimicrobial photodynamic therapy on Streptococcus mutans is altered by glucose in the presence of methylene blue and red LED. Photodiagnosis Photodyn Ther 2017;19:1–4. 28414082 10.1016/j.pdpdt.2017.04.004

[9] Belinello-Souza EL Alvarenga LH Lima-Leal C Antimicrobial photodynamic therapy combined to periodontal treatment: experimental model. Photodiagnosis Photodyn Ther 2017;18:275–8. 28330815 10.1016/j.pdpdt.2017.03.008

[10] Botta SB Ana PA Gonçalves MLL Photodynamic therapy associated with a Blue Dye Papain-based gel and evaluation of its degradation of Type i collagen fibers. Photomed Laser Surg 2018;36:100–4. 29023186 10.1089/pho.2017.4342

[11] Bussadori SK Avaliação in Vitro Do Potencial Antimicrobiano De Dois Sistemas Para Remoção Químico-Mecânica De Dentina Cariada: Carisolvtm E Papacárie ® in Vitro Evaluation of the Antimicrobial Activity of Two Materials Used for Chemical and Mechanical Removal of Cari. 2005;296–305.

[12] Maniezo De Sousa J Camillo Jordão M Gisette M Utilization of papain gel associated with atraumatic restoration technique in baby - clinical case report [in Portuguese]. Odontol Clín- Cient 2012;11:75–9.

[13] Silva ZS Huang YY De Freitas LF Papain gel containing methylene blue for simultaneous caries removal and antimicrobial photoinactivation against Streptococcus mutans biofilms. Sci Rep 2016;6:1–2. 27641507 10.1038/srep33270 PMC5027554

[14] Giannobile WV Braun TM Caplis AK Patient stratification for preventive care in dentistry. J Dent Res 2013;92:694–701. 23752171 10.1177/0022034513492336 PMC3711568

[15] Campos GN Pimentel SP Ribeiro FV The adjunctive effect of photodynamic therapy for residual pockets in single-rooted teeth: a randomized controlled clinical trial. Lasers Med Sci 2013;28:317–24. 22814896 10.1007/s10103-012-1159-3

[16] Chapple ILC Mealey BL Van Dyke TE Periodontal health and gingival diseases and conditions on an intact and a reduced periodontium: Consensus report of workgroup 1 of the 2017 World Workshop on the Classification of Periodontal and Peri-Implant Diseases and Conditions. J Periodontol 2018;89:S74–84. 29926944 10.1002/JPER.17-0719

